# A Model for Clinical Coder Satisfaction in Saudi Arabia Based on a Holistic Approach: Clinical, Professional and Organizational Dimensions

**DOI:** 10.7759/cureus.37966

**Published:** 2023-04-22

**Authors:** Abdullah T Alanazi, Eman Al Alkhaibari, Bakheet Aldosari

**Affiliations:** 1 Health Informatics, King Saud Bin Abdulaziz University for Health Sciences, Riyadh, SAU; 2 Research, King Abdullah International Medical Research Center, Riyadh, SAU; 3 Health Informatics, King Saud Ibn Abdulaziz University for Health Sciences, Riyadh, SAU

**Keywords:** holistic approach, satisfaction, computer-assisted coding system (cacs), encoder, clinical coding

## Abstract

The quality of clinical coding influences not only hospital revenue but also the quality and efficiency of healthcare services. Assessing the coders' satisfaction is essential to optimizing the quality of clinical coding. This mixed-method study used a qualitative approach to propose the study model while testing the model through a quantitative approach. The relevant variables of the satisfaction model were assessed through a survey targeting clinical coders across the country on a timely basis. Fourteen experts participated in establishing the model with three dimensions: professional, organizational, and clinical. Each dimension has its relevant variables. One hundred eighty-four clinical coders participated in phase two. 34.5% were male, 61% held a diploma, 38% had a bachelor's and above, and 49.7% worked in hospitals having fully electronic health records. We found that organizational and clinical dimensions strongly correlate with coders' satisfaction. Noticeably, the most influencing variables were the availability of coding policies and the computer-assisted coding (CAC) system. The results show that the model explains the satisfaction of the clinical coders, and organizational and clinical-related variables are crucial. Although gender-based differences exist, training (regardless of the training mode), coding policies, and the CAC system substantially influence coders' satisfaction. A significant stream of the literature supports these findings. However, attempting a holistic approach to assess coders' satisfaction and affecting coding quality is the added value of this study. Optimizing clinical coding practice requires organization-wide initiatives and policies to regulate coding practices and standards to promote the quality and timeliness of clinical documentation. Training is indispensable not only for clinical coders, but physicians also need to understand the rationale and value of clinical coding. Better utilization of the outcomes of the coding process and adopting the CAC system are significant drivers to enhance coders' satisfaction.

## Introduction

Clinical coding aims at converting healthcare service events into standardized and agreed-upon alphanumeric codes to represent those events. Each event is associated with specific services and is estimated to consume certain resources. Consequently, the clinical efforts and expenses are expected and recognized by different stakeholders of the healthcare services. Historically, causes of death were recorded in England in 1837 and prospered in the United States in 1933. Standardizing of causes of death was essential, as discrepancies were widely observed. As the need emerged, the International Classification of Disease (ICD) was designated by the United Nations to be managed by World Health Organization in 1946. WHO is responsible for managing ICD and updating and maintaining revisions. Using ICD is not meant exclusively for mortality purposes. Morbidity reimbursement for diseases and procedures along with standardizing the services and efficiency of resources are among the other purposes of ICD. 

Coding causes of Morbidity are even more critical for indicating specific clinical encounters. By providing a comprehensive list of all diseases and procedures, clinical coders must review clinical documentation, principal diagnosis, and lab and radiology results in patient charts to assign the correct ICD code. A clear and complete disease description is the only way to give a valid code. Further to assigning primary code, comorbidity is also tracked and recorded considering the intensity of care, and the associated cost of care could be influenced significantly. Assigning ICD codes determines the Diagnosis-Related Group (DRG), which represents the single payment healthcare facility will receive for each clinical encounter. Later, the severity scale was introduced to DRG to control the rising cost of healthcare services. Thus, ICD coding is a venue and basis to assess the quality of provided services and efficiency - which correlates with the intensity of services - and further as a means for third-party reimbursement. 

Clinical coding requires specific professional competencies in the aspect of knowledge and skills of coding practice. In addition to formal education, intense training for health information management professionals is needed to cope with the recent version of ICD, which is considered more clinical-oriented than previous versions of ICD.

Computer-Assisted Coding (CAC) uses Natural Language Process (NLP) technique to identify specific terminologies to indicate diseases and procedures from patient charts in the Electronic Health Record and then assign the proper ICD codes for diseases and procedures. Further, CAC can evaluate the context of designated terms to indicate whether such term is coded. Nevertheless, human coders must review the suggested codes generated by CAC to optimize the accuracy of the assigned code and the necessity of codes or assess the missing coding events. CAC helps clinical coders by enhancing their coding accuracy and productivity. Instead of the need to retrieve data from different modules and health information systems by the clinical coders, CAC is integrated into the workflow of these systems and can bring the relevant documents and information, allowing quicker access by the clinical coders. 

Many variables influence the quality of coding practice. In a recent paper, Shepheard mentioned poor quality of clinical documentation, keeping up with clinical case definition, and the necessity of capturing all comorbidity and preexisting conditions as the main obstacles for clinical coders [1]. The quality of clinical documentation mainly influences coding practice; as Farhan J et al. mentioned, "Major problems identified in coding were related to the quality of the medical record documentation rather than to the quality of the coding" [2]. To enhance documentation quality, physicians must be aware of clinical documentation requirements. For example, physicians may need more information and time to complete a discharge summary and make it reflect the patient's status during hospitalization and treatment [3]. Farhan et al. stated that only 60% of medical records met the proper coding and documentation in a tertiary hospital [2]. Another study found that cases with incomplete discharge summaries are 215 times at higher risk for incorrect coding [4]. 

As ICD coding is the basis for DRG assignment, Coders must capture all comorbidity and preexisting conditions. Variations based on diseases and comorbidity are observed. The intensity and complexity of health services have adversely impacted the assigned codes' quality. For example, diabetes and Crohn's disease have the highest improper assigned codes. Omitting secondary diagnoses is common [5]. 

Additionally, keeping up with the latest clinical case definitions is another struggle for clinical coders. Phillips and his collogues highlighted the inconsistency in assigning malnutrition code and the recent clinical malnutrition case definition. They questioned whether the assignment of E43 should continuously be revised or shall we follow a more stable process when coding and consider only updating case definition upon updating coding practice [6].

As coders have different qualifications and personal characteristics and this may influence the quality of coding practice. Further, training coders is considered among the most crucial variable in enhancing coding quality. A substantial literature has emphasized the importance of training both clinical coders and physicians [2,7,8]. Nevertheless, Hennessy et al. found that neither coders' characteristics nor coding sites influence the validity of ICD-10 codes [9].

On the other hand, Health Information Management (HIM) directors should ensure appropriate policies to support best coding practices [10]. Having proper policies would require adequate planning and preparation for ICD adoption and policies for the health information management departments, which are essential for successful coding practice [11]. Scenario planning is a process to adopt when a new version of the ICD is introduced or an initiative is undertaken to enhance the quality of clinical coding [7] Clinical scenarios are an attempt to resemble cases for example patient with heart disease or gastrointestinal system [12].

The Computer-Assisted Coding (CAC) system is believed to enhance the accuracy and quality of clinical coding [13]. However, inefficient CAC systems are considered one of the critical challenges for many healthcare institutions, as they have to offer the best level of integrated data within their electronic system to overcome their coding flaws [14]. However, Osborn and Zale, in their study in 1996, found that coders who code manually are more satisfied than those with encoder systems [15]. Correspondingly, vendors of the next generation of CAC must support exchanging data with other health systems [16]. Nevertheless, designing an appropriate interface for CAC systems based on professionals' recommendations is needed [2,7].

The recent transition to ICD-10 has added more burden on clinical coders, the considerable number of codes with detailed descriptions for each disease, and the already shortage of clinical coders. Furthermore, the coding error is obvious and negatively affects hospital revenue. A Malaysian study assessed the matching of assigned codes between two coders and found that coding errors happened in 89% of patient medical records and contributed to lower revenue in 52% of the cases [4]. In addition to the challenging nature of the job, clinical coders may be unhappy with their income, as stated in one market analysis report, clinical coding was in the bottom 16% of careers [17]. A recent study revealed that 37% of clinical coders are not happy with their salary, and 17% of current coders have the desire to resign. Also, the study disclosed a positive correlation between salary satisfaction and having a higher coding certificate, which is only attainable, as the study revealed [18]. Henceforth, there is a need to assess coders' satisfaction and examine the potential variables contributing to their perceptions and attitudes toward their job. Consequently, to provide recommendations to enhance coder satisfaction and quality of clinical coding. The impact of a coder's characteristics and clinical and organizational-related variables is assessed in this study.

## Materials and methods

This study used a mixed-method approach in which a qualitative study explores the relevant variables that influence the satisfaction of clinical coders. Then, a quantitative study assesses and tests the variable from clinical coders' perspectives.

An expert panel was conducted after a thorough literature review revealed poor coverage for the local variables determining coders' satisfaction. Seventeen experts were enlisted and approached. All the panelists have at least five-year experience in clinical coding and or hold a senior position in the medical record department with direct exposure to clinical coding. Therefore, they were selected purposively, and invitations were sent. During the panel, participants were encouraged to communicate and speak frankly to reflect on their ideas and opinions. Further, panelists were allowed to review their opinions, understand the consistency of their ideas with others, and, if necessary, restate their ideas to attain a consensus. The results of this phase should illustrate the relevant dimensions and variables in the proposed model to assess coders' satisfaction.

A cross-sectional descriptive design used open-ended questions to collect data to assess and predict coders' satisfaction. A convenient sample technique was used for clinical coders from Saudi Arabian hospitals. The survey was developed based on the results of phase one and is divided into four parts: socio-demographic characteristics of respondents and questions about the different dimensions that impact coders' satisfaction: Professional, Organizational, and Clinical. Fifteen questions were written (12 open-ended and three closed-ended questions). Each variable was aimed to be assessed by one question. Three questions about personal attributes data (Gender, Education, English Literacy). Three questions about the Healthcare and Medical Record Department (type of healthcare facility, type of medical record, whether using ICD for morbidity or just for morality). Next, eight questions for aspects around clinical coding (closed-end questions about training, reuse of coding data for further actions, and the existence of a national professional body, and three open-ended questions about policies regulating coding practice, current clinical documentation status, and the services or functions provided by your CAC System). Moreover, the final question is about the overall satisfaction levels.

Face and internal validity were assessed by three students of the health informatics master program and one faculty member. The questions were written in direct and plain language to clarify and eliminate ambiguity to measure the target variable directly. Next, a pilot study was conducted, and results indicate that questions could measure the intended objectives.

## Results

Phase one: experts panel 

Out of 17 panelists, 14 (82%) participated in the panel. Three participants left for personal reasons. The experts' panel had two rounds in which experts identified variables that may influence the daily work of clinical coders and their satisfaction in the first round. After enlisting all variables, experts were asked to rate them on a scale of 1 to 10 based on their importance to coders' satisfaction. In round two, all variables were compiled, and a consensus was built based on the agreement of 50% and more of the experts, and further, regrouping these variables in relevant dimensions. As a result, dimensions (with its variables) were taken to the next phase, in which the model was built and tested through a survey-based approach (Table 1).

**Table 1:** Variable Definition, Consensus, and Relevancy to the Assigned Dimension.

Dimensions and Variables		Definition	Consensus of the variable	Consensus of variable to dimension
Personal and Professions Factors associated with coders characteristics and qualification	Qualification	State of academic qualification and English literacy	93%	100.0%
	Professional body	Existence of supported professional body promoting best coding practice	57%	64.3%
Organizational Organization and Medical Record department related variables	Coding Policies	Guide coding practices to achieve outcomes that reflect best coding practice	85.7%	100.0%
	Enabling of reuse of coding data	Utilizing the outputs of coding data for further actions and analysis	71.4%	78.6%
	Training	Existence and type of coding training courses	100%	64.3%
Clinical Encounter Factors associated with the clinical documentation and encoding within the information system	Encoding system	Assess the ability of Encoding system integrated in health information system (HIS) and facilitates coding practice.	71.4%	64.3%
	Clinical Documentation	Assess if the clinical documentation about the state of the patient supports the quality of coding.	100%	100.0%
Satisfaction	Definition			
	Level of coder satisfaction about current coding practice			

Phase two: survey results

The study was conducted in secondary and tertiary healthcare facilities across Saudi Arabia, and the targeted population is clinical coders, particularly those who code using the International Classification of Disease (ICD-10). Two hundred and nine responses were received from participants from different Saudi Arabian hospitals. However, 25 questionnaires (12%) were excluded from further analysis due to incomplete and missing data (<50% of questions were not answered). The final sample size was 184 participants with a response rate of 83.6%. 72 (34.5%) of the participants were male, 127 (61%) held a diploma, 72 (34.5%) had a bachelor's, and 3.4% had a master's and above. 113 (54%) of the participants' English competency level was intermediate, 61 (29%) advanced, and the remaining were elementary level. 140 (67%) of the participants worked in secondary hospitals, while the remaining were in tertiary hospitals. Among those surveyed, 75 (36%) of their hospitals had hybrid health records (both electronic and paper-based); 104 (49.7%) had fully electronic health records (EHR). As a coding practice, 173 (83%) of the participants used ICD for morbidity purposes, while 17% used ICD codes only for mortality. Regarding the training received, 134 (64%) mentioned face-to-face training, 12% took online courses, and 20% received no formal training. Only 138 (66%) of the participants knew the availability of coding courses. Regarding the efficacy of internet-based training, 136 (65%) believed that internet-based could be an effective means to get proper training in ICD coding.

Next are the responses to the open-ended questions; thematic analysis for questions regarding the coding policy, clinical documentation, and CAC are presented in Table 2.

**Table 2:** Thematic analysis for the three open-ended questions regarding coding policy, clinical documentation, and CAC CAC: computer-assisted coding

Measure	Item	Percentage%
Policies regulate Coding	Incomplete Diagnosis	5%
	Coding Process, Incomplete Diagnosis, Terminology Standards and Compliance	29%
	No policy exists	14%
Clinical Documentation	Unspecified Diagnosis	7%
	Lack of Proper Discharge Summary	13%
	Lack of Proper Discharge Summary, and Unspecified Diagnosis	33.3%
	Lack of all proper documentation aspects	10.7%
Encoding System CAC	Support Coding Practice	29.4%
	User-Friendly Interface	22.6%
	Ability to monitor healthcare data	22.6%
	All services/functions are presented	6%
	All services/functions are not implemented	34.5%

Regarding the availability of policies regulating the coding practice, the responses were grouped through thematic analysis as followings: 14% of the participants revealed that they have no policy at all; 5% said that they have only policy regulating incomplete diagnosis; 29% of the participants acknowledged having policies for the following: coding process, incomplete diagnosis, terminology standards, and coding compliance.

Regarding the impact of current documentation on the quality of coding, the thematic analysis revealed that 33.3% of the participants suffered from the following: lack of proper discharge summary, lack of determining primary and secondary diagnosis and having no specified diagnosis. 13% of the participants acknowledged that a lack of adequate discharge summary was the main documentation issue. Further, 7% believed that only unspecified diagnosis was their main struggle. On the other hand, 10.7% revealed that all poor documentation issues presented and influenced their coding quality significantly.

Remarkably, 29.4% of the participants valued the importance of CAC within their health information systems to support coding practice. 22.6% mentioned that their CAC has a user-friendly interface; similarly, 22.6% said that their CAC could monitor the quality of their data. However, 34.5% of the participants indicated that most of the needed functions of CAC still need to be implemented in near future, and only 6% of the participants stated that they have all the required functions in their CAC.

On the other hand, 42% of the participants have emphasized that the results of clinical coding are not utilized for clinical decisions and that their hospitals do not reuse the assigned codes for further actions. In contrast, only 27.4% indicated that the hospital is taking further action. On the other hand, 90.5% of the participants believed that having a national coding body to oversee coding would improve the coding profession and practice.

The last question aimed to measure overall satisfaction; 52.3% of the participants were satisfied, and 41.7% were dissatisfied. At the same time, 6% rated themselves as neither satisfied nor dissatisfied.

For inferential statistics, there is a statistically significant difference between females and males in their satisfaction levels, as females were less satisfied than males (t= -2.2., p<0.05).

Generally, the coding training, type of medical records, availability of policies, the impact of electronic medical records (EMR), and utilization of assigned codes for further action revealed statistically significant differences in coders' satisfaction levels. The responses to questions related to the existence of the coding policy, clinical documentation, and functions of the CAC system were recoded to enable further analysis.

The professional dimension (Education, English Literacy, Existence of Professional Body) had no statistically significant impact on coders' satisfaction (R Square=.066, F=1.391, P=0.2). Only the gender variable affected satisfaction. 

The organizational dimension (utilization of ICD, availability of coding courses, training type, the efficacy of internet-based training, hospital type, medical record type, and availability of policies) was found to influence coders' satisfaction (R Square=0.2, F=2.73, p=0.01). The post hoc analysis shows that satisfaction level is higher with coders who work in hospitals that take coding outcomes for further action. Higher satisfaction levels were found with coders who work in an organization with a coding policy and have received training, and coder who works in hospitals with EHR compared to the counterpart groups. The implementation of policies was the most important variable in this dimension.

The clinical dimension (clinical documentation, functions of CAC system) has a strong significant effect, and the strongest influence is found in the "Impact of Encoding System". See Table 3.

**Table 3:** Most influencing Variables in Models.

Model	Variable	B	Std. Error	Sig
1	Gender	.989	.479	.04
2	Availability of Coding Policies	.05	.021	.01
3	Impact of Encoding System	.151	.039	.00

## Discussion

Undoubtedly ICD coding helps healthcare facilities to measure the quality and efficiency of their services, as it is a means for standardizing care and a basis for reimbursement [19]. Nevertheless, adopting ICD coding has been slow, and still, some hospitals need help to catch up, especially charity and governmental hospitals that do not use ICD for reimbursement. Alkraiji et al. found that many coding systems and standards were not adopted for technical, managerial, and educational reasons [20]. Therefore, this study aimed to propose a model to address satisfaction based on three dimensions: profession, organizational, and clinical.

The results of the variables of the profession dimension reveal no statistically significant differences in coders' satisfaction, except for gender, as males are more satisfied than females. The gender differences in our study can be attributed to the dominance of females in the coding profession, and further, they usually outperform males in academic performance; as one study stated, thereby they may have higher expectations than males, and this would result in less satisfaction in case of any flaws [21]. These findings follow the study of Hennessy et al., which indicated that coders' characteristics do not influence coding practice [9].

The organization dimension is associated with increased satisfaction of clinical coders. The analysis indicated a statistically significant difference in coders' satisfaction based on the type of medical record, availability of training courses, coding policies, and utilization of ICD outcomes for further actions. Prior works have documented the role of training in enhancing coders' skills [2,7,16]. Rousse, in his study, found that education is a significant factor, and coder must advance their knowledge, critical thinking, and problem-solving skills to move from novice to expert coder [16]. Thus, training is essential to bridge the gap in coders' knowledge and skills and address challenges in assigning proper ICD codes. Our study found that coders are influenced by the availability of coding courses rather than the methods of delivering the training (face-to-face or online). These findings comply with other studies. Otero Varela and her collogues conducted an international survey in 2022 to assess the availability of coding training, resources, and length of the training, and revealed that the required coding training exists in Saudi Arabia [22]. However, there needs to be more on-job training and continued education in clinical coding in Saudi Arabia.

The results of our study unveil that implementing appropriate coding policies enhances the satisfaction levels of coders and improves coding practice. This finding complies with the study of Otero Varela et al. which revealed that lacking coding standards have negatively impacted the quality of clinical coding [23].

Additionally, there is a statistically significant difference regarding utilizing ICD outcomes for decision-making factors and the satisfaction level of coders, as coders who work in hospitals that take coding outcomes for further actions tend to be more satisfied than their counterparts. Relatedly, setting up performance metrics and initiating policy are major drivers for better practice [24].

The last dimension is clinical, and we found that the clinical dimension statistically influences coder satisfaction. Nevertheless, the responses indicate that the quality of documentation did not influence coders' satisfaction as much as the availability of the CAC system. Unquestionably, clinical coding quality depends on clinical documentation quality [23,25]. However, the undervalued role of clinical documentation can be attributed to the need for national standards for clinical documentation. The lack of requirements for data collection is a global concern, as mentioned by the study of Otero Varela and others in 2021, which revealed that high and low-income countries need more proper standards for clinical documentation in place [23]. Also, one study found that most healthcare systems worldwide capture and focus only on diagnosis fields [26]. The number of fields for diagnosis positively correlates with the quality of clinical coding, as a reduced number of fields is associated with undercoding [23]. The CAC system is automated, can support coding processes and can monitor healthcare data. These items highlighted and provided compelling evidence that the CAC system influences coders' satisfaction. The study by Otero Varela et al. in 2021 mentioned that around 30% of surveyed clinical coders worldwide prefer having a CAC system as a supporting tool during coding [23]. Campbell and Giadresco conducted a study to assess coders productively, revealing that CAC systems have significantly enhanced coders' productivity [13]. Although some of the participants work in facilities with paper-based records, participants who work in electronic records expressed their desire for an appropriate CAC system to resolve some medical coding problems. These problems are represented in poorly retrieved data that cannot enhance coding and decision-making and a need for proper integration between the available electronic systems in their facilities. The results of Campbell and Giadresco's study go along with our results that today's CAC systems' functions, accuracy, and usability have improved coding practice, influencing coders' jobs [13].

Recommendations

For an optimal clinical coding practice, it is important to ensure the engagement of top leaders and other managers to support and smooth the endeavor. In the beginning, conduct a gap analysis for available and needed resources. Seek expert consultation to determine priorities, investigate options, and focus on opportunities through developing a strategic plan for enhancing coding practices. Then seek best practices recommendations, propose policies based on governmental regulations and third-party payers, and set a monitoring process for applying these policies periodically to identify any flaws and deviations. Additionally, provide sources such as books, software, and internet-based resources that aid during daily coding practices. For training courses, all coders must receive training to enhance their quality of coding practice. However, the need for more coders can be overcome by outsourcing part of the work and adopting sophisticated CAC systems. Determine the deficiencies in clinical documentation, target the physicians or departments with customized courses, and identify what coding is and why it is important to have proper clinical documentation. Identify the approved abbreviations that must be followed. Take advantage of the outcomes of coding practice, such as statistical information about diseases and patients, previous treatments for similar conditions, and the possibility of conducting research, and communicate these results in graphs or dashboards. This method helps physicians and facilities to activate the reusing of coding outcomes. Furthermore, apply policies to incentivize physicians to ensure timeliness of the discharge summary completion. In the end, constantly monitor coding practice, analyze the deviations, and celebrate success.

Although this study was conducted through two phases, it has exploratory and descriptive nature in which data were collected through an expert panel and a survey-based approach in the second phase. Making conclusions and generalizing the findings of this study over other settings should approach cautiously.

## Conclusions

More than ever, clinical coding is pivotal in today’s healthcare facilities - they must monitor their coding practices and identify critical success factors that can influence the accuracy of clinical coding. Coders’ satisfaction is essential to coding practice quality and retaining coders. The proposed satisfaction model of clinical coders was built based on three dimensions: professional, organizational, and clinical encounters. Noticeably, our results found a shred of compiling evidence that both organizational and clinical dimensions are highly associated with the satisfaction of codes. The results demonstrate that the most important variables are the availability of coding policies and CAC systems integrated within any healthcare facility's workflow and other information systems. At the end of the study, recommendations are presented for properly managing clinical coding.
